# Cellulose-Based Composite Hydrogels for Heavy Metal Ion Removal: Recent Advances and Engineering Perspectives

**DOI:** 10.3390/gels12050380

**Published:** 2026-04-30

**Authors:** Xiaobo Xue, Jihang Hu, Panrong Guo, Liyun Wang, Luohui Wang, Youming Dong, Fei Xiao, Cheng Li, Shen Ding

**Affiliations:** 1 College of Forestry, Henan Agricultural University, Zhengzhou 450002, China; 2 College of Materials Science and Technology, Nanjing Forestry University, Nanjing 210037, China; 3 Research Institute of Wood Industry, Chinese Academy of Forestry, Beijing 100091, China; 4 Hunan Academy of Forestry, Changsha 410018, China

**Keywords:** cellulose, hydrogel, heavy metal adsorption, pollution remediation, sustainable development

## Abstract

With the rapid intensification of industrial and agricultural activities, water contamination by heavy metal ions has emerged as a critical global challenge, gravely imperiling ecosystem stability and public health. Among the various remediation technologies, adsorption has been widely adopted due to its high efficiency, low-cost water treatment, and simplicity of operation. However, conventional inorganic or synthetic adsorbents often exhibit poor degradability and pose a risk of secondary contamination, substantially limiting their sustainable application. Consequently, the development of environmentally benign and renewable adsorbent materials has become a central research focus in this field. Recently, cellulose-based composite hydrogels, derived from renewable resources and characterized by excellent eco-friendliness and highly tunable three-dimensional porous structures, have attracted considerable attention as promising green adsorption materials. These hydrogels demonstrate outstanding performance in the efficient sequestration of heavy metal contaminants from aqueous environments. This review systematically summarizes recent advances in cellulose-based composite hydrogels for heavy metal removal, to elucidate the structure–performance relationships linking material fabrication strategies, structural modulation, and adsorption efficiency. First, we outline the principal construction approaches, including physical crosslinking, chemical modification, and supramolecular self-assembly, and comprehensively analyze how different synthesis routes regulate pore architecture, mechanical properties, and the distribution of surface functional groups. Second, the underlying adsorption mechanisms, primarily coordination complexation, electrostatic interactions, and ion exchange, are discussed in detail. Finally, recent studies on the adsorption of cationic heavy metals (e.g., Pb(II), Cu(II), and Cd(II)) and anionic oxyanions (e.g., As(III) and Cr(VI)) are critically reviewed, with particular emphasis on the relationships between selective adsorption performance, material design principles, and specific recognition mechanisms. Overall, this review provides a theoretical foundation and practical guidance for the design and development of next-generation water treatment materials with high adsorption capacity, excellent selectivity, non-toxicity, and strong environmental compatibility, followed by future research recommendations.

## 1. Introduction

Global industrial wastewater discharges have reached an alarming scale, exceeding 30 billion tons annually, with a significant portion of this volume containing heavy metals [1]. Among these contaminants, heavy metal ions such as lead (Pb(II)), copper (Cu(II)), cadmium (Cd(II)), arsenic (As(III)), and hexavalent chromium (Cr(VI)) pose persistent and severe threats to ecosystems and human health owing to their high toxicity and bioaccumulative behavior [2,3,4]. In fact, these heavy metals cause serious threats with severe diseases, i.e., kidney failure, liver cancer, anemia, and high blood pressure, as well as foot and mouth disease. Consequently, effectively eliminating heavy metals from wastewater to discharge would benefit environmental benignity, ecological risks, and remediation. These heavy metals are also recognized as “priority pollutants” predominantly generated from distinct, large-scale industrial waste streams. For instance, electroplating and printed circuit board (PCB) effluents are notoriously rich in Cu(II) and Cr(VI); battery manufacturing and acid mine drainage are primary sources of Pb(II) and Cd(II); while semiconductors and smelting effluents frequently contain persistent As(III). Furthermore, in practical industrial scenarios, these heavy metals rarely exist individually; they are typically present simultaneously, creating complex multi-component wastewater matrices. In such coexisting systems, the adsorption process is governed by competitive dynamics, where different metal ions compete for specific binding sites based on their ionic radii, electronegativity, and coordination affinities (e.g., Pearson’s Hard–Soft Acid–Base theory). Conventional treatment technologies suffer from notable drawbacks. Chemical precipitation typically requires substantial alkaline reagents and generates difficult-to-manage hydrous sludges. Ion-exchange resins incur high regeneration costs and are highly susceptible to interference from coexisting ions in complex aqueous matrices. Activated carbon, although widely used due to its high adsorption capacity, generally exhibits limited selectivity and struggles to achieve precise capture of target heavy metal ions [5,6,7]. In light of the economic and environmental constraints associated with these traditional physicochemical approaches, increasing attention has been directed toward bio-based adsorbents. Owing to their low cost, sustainability, excellent adsorption capacity, and environmental compatibility, these materials have emerged as promising alternatives and represent a significant advancement in the remediation of heavy metal-contaminated wastewater.

Among various biomass-derived materials, cellulose stands out as the most abundant natural renewable polymer on Earth, featuring remarkably widespread raw material sources and an estimated annual production of approximately 1.5 × 10^12^ tons [8,9,10]. Beyond its abundance, the molecular structure of cellulose offers exceptional chemical versatility. Each anhydroglucose unit in the cellulose molecular chain contains three hydroxyl (–OH) groups, providing a high density of reactive sites that are amenable to chemical functionalization [11]. For instance, TEMPO-mediated oxidation introduces carboxyl (-COOH) groups, thereby enhancing heavy metal adsorption via coordination complexation. Grafting amino (-NH_2_) or thiol (-SH) groups significantly enhances specific coordination to “soft acid” metal ions such as Cd(II) and Pb(II), while phosphorylation modification endows materials with selective recognition for radionuclides such as uranium [12,13,14]. Despite its chemical richness, natural cellulose typically exists as micro/nanopowders; this morphology leads to buried active sites, high mass transfer resistance, and difficult recovery [15]. Assembling cellulose into monolithic three-dimensional (3D) hydrogels offers a transformative solution; it not only overcomes these practical bottlenecks but also establishes a fundamental structural advantage over benchmark adsorbents such as functionalized activated carbon (AC) and emerging AC-based composites. Functionalization strategies are widely applied to AC for selective capture. However, chemical grafting on its rigid carbon framework—or the introduction of secondary polymer binders in AC composites—inevitably restricts internal micropores and severely hinders ion diffusion [16]. In contrast, pure cellulose inherently forms a seamlessly integrated, highly hydrophilic 3D network. This robust skeleton remains fully expanded in water, ensuring unobstructed intra-particle channels that enable ultrafast adsorption kinetics. Moreover, the inherent abundance of surface hydroxyl groups provides high-density anchoring sites for functionalization under mild conditions. Additionally, its cohesive macroscopic architecture enables straightforward post adsorption separation, eliminating the persistent risk of secondary microparticle pollution, for instance, (leaching) associated with granular/composite AC and powdered materials. Consequently, rational engineering of cellulose molecules and their assembly into macroscopically controllable bulk materials is essential for overcoming these limitations and enabling efficient, scalable application in water treatment.

Recently, cellulose-based composite hydrogels have emerged as a robust solution to mitigate these engineering constraints. Unlike macroplastic beads, a common alternative that offers high rigidity for packed bed columns, these hydrogels avoid the inherent densification that severely restricts internal ion diffusion and buries active sites in bead structures. [17]. In contrast, by constructing well-defined three-dimensional network architectures, hydrogels completely avoid the structural densification typical of macroplastic beads. Such materials achieve a high density of accessible active sites at the solid–liquid interface, enabled by their ultrahigh water contents (>90%). Simultaneously, their interconnected hierarchical pore channels, typically in the range of 10–100 μm, function as efficient ion transport pathways, enhancing diffusion kinetics by several orders of magnitude relative to those observed in dense membrane-type materials or rigid beads [18,19,20]. Although the ultra-high porosity of monolithic hydrogel inevitably yields lower compressive stiffness under high-pressure conditions compared to beads, their monolithic format fundamentally resolves the separation difficulties associated with nano- or microscale powder adsorbents. From an engineering and practical application perspective, although the ultra-high porosity inevitably yields a lower compressive stiffness under high-pressure conditions compared to beads, the monolithic hydrogel format fundamentally resolves the separation difficulties associated with nano- or microscale powder adsorbents. Solid–liquid separation can be readily achieved via simple mechanical compression, avoiding the need for energy-intensive centrifugation [21,22,23]. Furthermore, a hierarchical synergistic system can be established by incorporating secondary biopolymers (e.g., chitosan or sodium alginate) to construct double network hydrogels, or by integrating nanoscale functional components (e.g., graphene or metal organic frameworks, MOFs) in situ. This hierarchical design features “macroscopic skeleton support–microscopic pore transport–molecular level site capture”, enabling either broad-spectrum capture or highly selective removal of complex heavy metal contaminants, depending on the tailored active sites [24,25,26].

Despite the rapid expansion of research on cellulose-based hydrogels, which has revealed multiple adsorption mechanisms involving electrostatic interactions, coordination complexation, and ion exchange [27,28,29], most reported advances remain limited to laboratory-scale performance evaluations. Several critical challenges still impede practical implementation. These include the following: (i) achieving precise regulation of hierarchical material structures while maintaining high adsorption capacity; (ii) clarifying the competitive adsorption behavior and synergistic interactions among multiple coexisting pollutants in complex aqueous environments; and (iii) bridging the gap between laboratory-scale materials and stable, scalable water treatment processes. In this contest, the present review systematically surveys recent progress in cellulose-based composite hydrogels for heavy metal remediation. Particular emphasis is placed on elucidating the regulatory principles by which fabrication strategies govern microstructural features and macroscopic adsorption performance. In addition, the adsorption behaviors and underlying mechanisms of cellulose-based composite hydrogels toward different heavy metal species are comprehensively summarized. Finally, perspectives are provided on their future research directions via application potential, key technical bottlenecks, and challenges associated with scaling up for wastewater valorization and practical treatment systems.

## 2. Fabrication Methods for Cellulose-Based Composite Hydrogels Oriented for Adsorption

The physicochemical properties of cellulose-based composite hydrogels fundamentally depend on their synthesis pathways. Whether through physical crosslinking, chemical crosslinking, or self-assembly methods, the core principle involves precisely tuning the crosslinking density, porosity, and distribution of functional groups within the three-dimensional network. This enables the customization of the mechanical modulus, swelling behavior, and mass transfer kinetics, ultimately determining the adsorption capacity and selectivity for specific heavy metal ions.

### 2.1. Physical Crosslinking Methods

**Figure 1:**
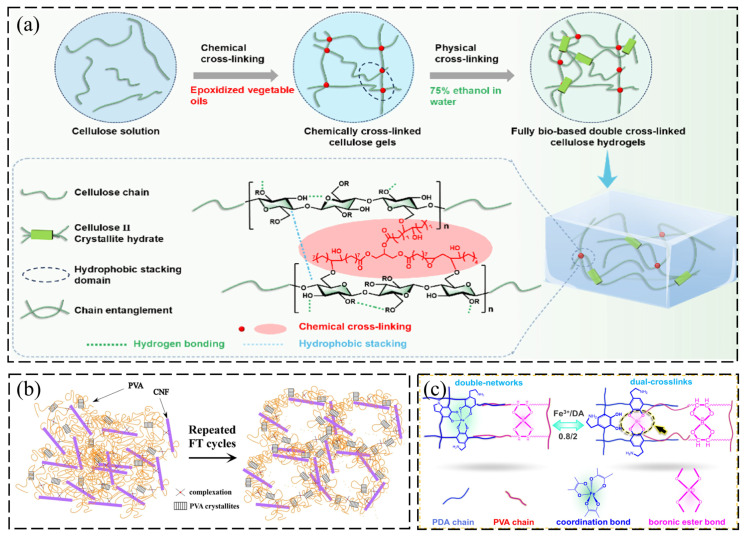
(**a**) Schematic illustration of the preparation process for high-strength FBDC cellulose hydrogels. Reproduced with permission from reference [32]. Copyright 2024, American Chemical Society. (**b**) Schematic representation of the effect of freeze–thaw (FT) cycles on composite hydrogel structure. Reproduced with permission from reference [33]. Copyright 2024, Springer Nature. (**c**) Schematic diagrams of characteristic network structures in PVA/CNF ionic hydrogels with varying Fe(III)/DA ratios. Reproduced with permission from reference [35]. Copyright 2023, Elsevier.

Physical crosslinking methods construct hydrogel networks through non-covalent forces such as hydrogen bonding, electrostatic interactions, chelation, and microcrystalline formation [30]. Among these approaches, freeze–thaw cycling-induced molecular chain rearrangement not only offers mild processing conditions but also eliminates residual chemical crosslinking agents, making it highly compatible with the fabrication requirements of environmentally friendly adsorbent materials [31]. However, the relatively weak bonding energy of physical crosslinks often leads to limited mechanical properties. The schematic diagram shows the formation mechanisms and structural analysis of cellulose hydrogels via chemical and physical crosslinking methods, and contains three panels: (i) synthesis pathways, (ii) microscopic structure, and (iii) molecular interactions, as delineated in Figure 1a–c. As shown in (Figure 1a), on the left side, the cellulose solution (dissolution stage) is followed by the middle stage of the chemically crosslinked cellulose gels using equilibrium vegetable oil emulsion, and the left side of the diagram illustrates the physically crosslinked gels (75% diffused in water) leading to fully bio-based double crosslinked cellulose hydrogels. The diagram delineates the molecular representations of the cellulose chain, cellulose II crystalline layer structure, hydrophobic stacking domains, and chain enlargement. The green dotted lines represent hydrogen bonding; red regions represent chemical crosslinking, and blue regions represent hydrophobic stacking (Figure 1a). A previous study [32] reported that this shortcoming can be effectively overcome through the introduction of nanofillers or interpenetrating networks (IPNs) in composite material design. Takeno et al. [33] developed synergistic dual networking in a cellulose nanofiber CNF/poly(vinyl alcohol) (PVA) system via borax-assisted crosslinking and multiple freeze–thaw cycles. To summarize the impacts of repeated FT cycles on the hydrogel structure, the elevation in FT induced a crystallization of the PVA polymer of plastic and a porous arrangement of the polymer-poor, highly abundant, and rich phase, as delineated in (Figure 1b). Driven by the interplay of PVA crystallization, hydrogen bonding, and borate complexation, the resulting hydrogel achieved a strength of 2 MPa with an elongation at break exceeding 1000%. Similarly, Abe et al. [34] demonstrated the physical reinforcement effect of CNF, revealing that the addition of merely ~1.5 vol% CNF enabled the formation of an interpenetrating network structure through various physical entanglements and hydrogen bonding interactions with PVA chains, thereby enhancing the hydrogel’s Young’s modulus by approximately 250%. In addition, (Figure 1c) shows the crosslinking between the double networks, and the dual crosslinks are interconnected with the chemical Fe^3+^/DA ratio coordination complex and polymer chain interactions. The pink, purple, and red colors represent the crosslink junction of their interactions. An optimized composite hydrogel is suitable as a strain sensor for heavy metals removal with different crosslinks. In the context of heavy metal remediation, such physically reinforced structures are crucial, as they maintain structural integrity and prevent adsorbent loss even under the violent agitation typical of wastewater treatment tanks.

### 2.2. Chemical Crosslinking Methods

Unlike physical crosslinking, chemical crosslinking constructs robust covalent bonds, making it highly preferred for treating harsh, highly acidic, or corrosive heavy metal effluents (e.g., acid mine drainage) where weaker bonds would fail. The fundamental principle utilizes crosslinking agents (such as epichlorohydrin or diisocyanates) to achieve two objectives simultaneously: (i) enhancing structural weatherability, and (ii) introducing additional heteroatoms (O, N, S) that act as active adsorption sites for heavy metals [35,36,37]. These interactions impart exceptional stability to the hydrogels while enabling the precise tailoring of key properties, such as swelling capacity and mechanical stiffness. Yang et al. [38] employed epichlorohydrin (ECH) to crosslink cellulose and incorporated polyacrylamide (PAAm) to construct a dual-network hydrogel, leveraging the synergistic effect of dense crosslinking between ECH and PAAm to achieve an 11.5-fold enhancement in hydrogel modulus. This significant mechanical enhancement is primarily attributed to ECH crosslinking, effectively increasing the intermolecular distance between cellulose chains, while also benefiting from the rigid support provided by the dense structure formed by the PAAm network. Furthermore, Aswathy et al. [39] validated the use of green crosslinkers, utilizing the multiple carboxyl groups of citric acid to esterify the hydroxyl groups on carboxymethyl cellulose (CMC). Notably, beyond stabilizing the three-dimensional network, this specific citric acid-based crosslinking process leaves abundant unreacted carboxyl groups (-COOH). These groups act as excellent binding sites, significantly boosting the selective complexation capacity for target ions like Cd(II) and Pb(II).

### 2.3. Self-Assembly Methods

Distinct from physical crosslinking—which typically relies on external stimuli (e.g., freeze–thaw cycles) to drive macroscopic macromolecular entanglement—self-assembly methods leverage non-covalent forces such as hydrogen bonding and electrostatic interactions to spontaneously form highly ordered cellulose networks without external intervention. While physically crosslinked hydrogels prioritize mechanical reinforcement through forced chain rearrangement, self-assembly achieves structural ordering without requiring any external triggers or crosslinking agents, thereby preserving the material’s excellent intrinsic eco-friendliness [40,41,42]. This “bottom-up” construction strategy naturally generates hierarchical pore structures, which, due to their high surface area and multiscale porosity, exhibit tremendous potential for fabricating high-performance environmental remediation platforms. For instance, Zhang et al. [43] utilized electrostatic self-assembly of oppositely charged nanofibers, which prevents dense packing and thus constructs three-dimensional networks with an ultra-high specific surface area (54 m^2^/g). This highly exposed porous architecture maximized the contact probability between active sites and metal ions, which enabled remarkable adsorption capacities of 217 mg/g for As(III) (and simultaneously 531 mg/g for co-existing organic dyes like methylene blue). Notably, these adsorbents retained over 95% of their initial adsorption capacity after five regeneration cycles during which absorbed MB was stripped by immersing the BHA in 15 mL of 0.05 M HCl and shaking at 200 rpm and 25 °C for three 2 h cycles. This demonstrates that structurally optimized self-assembly can withstand the acidic desorbents required for heavy metal recovery, making it a sustainable candidate for water purification.

## 3. The Adsorption Mechanism of Cellulose-Based Composite Hydrogels

The removal of heavy metal ions by cellulose-based hydrogels is a complex process driven by the interplay of physical adsorption and chemical capture, characterized by the synergistic coupling of multiple interaction forces rather than single mechanism dominance. The main adsorption mechanisms are (1) electrostatic interactions, (2) hydrogen bonding, (3) coordination complexation, and (4) physical adsorption and pore entrapment. Specifically, the naturally abundant hydroxyl and carboxyl groups in cellulose endow the material surface with negative charges, which generate Coulombic attraction as a primary force for adsorption. This mechanism serves as one of the driving forces behind the efficient yet inherently non-selective (broad-spectrum) capture of various cationic heavy metals [44,45]. Introduction of amino groups (e.g., polyethyleneimine (PEI) grafting) for cationic modification can reverse the surface potential, achieving efficient removal of anionic pollutants such as arsenate and chromate [46]. The efficacy of this mechanism is significantly regulated by surface charge density and solution pH (which influences the protonation state of functional groups) [47,48]. (2) Hydrogen bonding: Although hydrogen bonds are relatively weak non-covalent interactions, they play an auxiliary anchoring role during the initial adsorption stage. Hydroxyl groups on cellulose chains can form hydrogen bond networks with metal ions and their hydration shells [49]. These ubiquitous interactions not only facilitate interfacial enrichment of ions but also serve as key factors in maintaining the stability of the hydrogel’s three-dimensional network [50,51]. (3) Coordination complexation: Unlike the broad-spectrum capture driven by electrostatic forces, this mechanism achieves highly selective chemical adsorption through specific ligand–metal bonding interactions. For transition metal ions such as Cu(II), Ni (II), and Co (II), lone-pair electron donors in the hydrogel (e.g., oxygen in -COOH, nitrogen in -NH_2_) act as ligands, undergoing strong coordination reactions with vacant metal orbitals to form stable chelates [52]. Compared to physical adsorption, chemical complexation exhibits stronger binding forces and excellent resistance to environmental interference. Introduction of chelating ligands (e.g., iminodiacetic acid, ethylenediamine, Schiff bases) represents an effective strategy to enhance this mechanism [53]. (4) Physical adsorption and pore entrapment: The porous structure of cellulose hydrogels provides more adsorption sites for metal ions [54], while the interconnected internal pore channels allow ions to diffuse into the material’s interior, thereby maximizing adsorption capacity. Nanofibrillation strategies significantly increase active sites by substantially enhancing specific surface area [55,56].

## 4. Adsorption Performance of Cellulose-Based Hydrogels Toward Different Heavy Metal Ions

Owing to their exceptional biodegradability and inherent renewability, cellulose-based hydrogels have emerged as promising candidates for heavy metal remediation. Current research focuses on systematically enhancing adsorption capacity, selectivity, and cycling stability by precisely controlling key parameters such as the three-dimensional network structure, surface functional groups, and crosslinking density. Current molecular design strategies for cellulose-based hydrogel adsorbents predominantly focus on targeted functionalization—that is, directional introduction of active sites such as carboxyl, amino, or phosphate groups onto the cellulose backbone, thereby significantly enhancing the material’s capture capability [57,58]. Common heavy metal ions, including Pb(II), Cu(II), Cd(II), As(III), and Cr(VI), can all be efficiently removed through specific binding with surface functional groups.

### 4.1. Adsorption of Pb(II)

To address the intrinsic deficiencies of cellulose hydrogels, including weak binding sites, poor mechanical properties, and difficult recovery, functionalization and modification strategies offer effective pathways for enhancing Pb(II) adsorption efficiency. Researchers achieve this through three complementary approaches: (1) introducing highly active coordination groups (e.g., amino, phosphonate) that utilize multiple synergistic mechanisms to amplify chemisorption affinity; (2) optimizing porous architecture and mass transfer kinetics via nanofillers (e.g., MXene, and graphene); and (3) resolving solid–liquid separation challenges through magnetic functionalization [59,60,61,62,63]. This hierarchical approach—from molecular-level site design to macroscopic structural regulation—achieves simultaneous enhancement of material adsorption capacity, rate constants, and cycle life.

**Figure 2:**
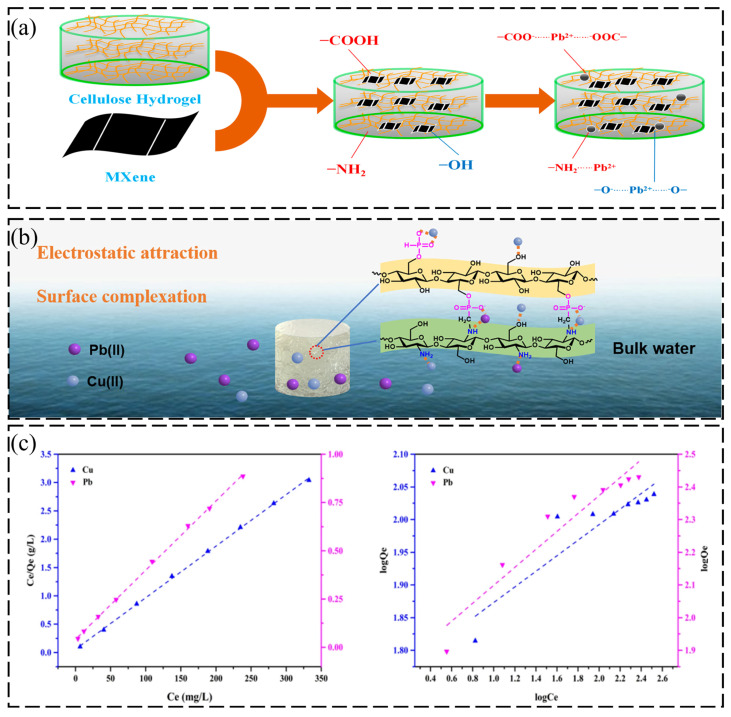
(**a**) Adsorption mechanism of Pb(II) by Mxene/cellulose hydrogel. Reproduced with permission from reference [59]. Copyright 2024, MDPI. (**b**) Preparation process of CS/MCCP and adsorption mechanism of CS/MCCP for Pb(II) and Cu(II). Reproduced with permission from reference [62]. Copyright 2024, Elsevier. (**c**) Langmuir and the Freundlich adsorption isotherm models for TCP adsorption of Cu(II) and Pb(II). Reproduced with permission from reference [63]. Copyright 2021, Springer Nature.

The development of superior adsorbents increasingly relies on utilizing inorganic nanomaterials to harness the power of multi-component synergy. Yang et al. [59] employed an in situ polymerization strategy to incorporate MXene (Ti_3_AlC_2_) into a cellulose matrix. MXen has important and efficient absorbent, chemical stability, and hydrophilicity that is advantageous for the adsorption of different molecules, such as heavy metals, organic pollutants, dyes, ammonia, and others. (Figure 2a) illustrates the schematic preparation of MXene/cellulose composite hydrogel and coordination between -COOH, -NH_2_, -CO-NH_2_ groups on the composite gel with Pb^2+^. The material achieved a remarkable adsorption capacity of 410.57 mg/g, attributed to the dual mechanism of physisorption and chemisorption enabled by the rich surface active sites of MXene nanosheets. Expanding on this synergistic paradigm, Shen et al. [60] fabricated an Fe-modified nanocellulose/sodium alginate/carrageenan composite system. Through a dual mechanism involving Ca (II)-mediated crosslinking and chemical anchoring via multi-functional groups (hydroxyl/carboxyl), this architecture not only delivered a high uptake of 351.04 mg/g but also ensured robust structural integrity over five regeneration cycles (Pb(II)-loaded AlKFc was desorbed with 20 mL of 0.1 M HCl at 298 K). Beyond compositional tuning, the topological optimization of the physical architecture proves equally critical. Adopting a distinct structural strategy, Kalaiselvi et al. [61] utilized a CaCO_3_ templating method to engineer carrageenan/cellulose hydrogels featuring 1–10 μm hierarchical porosity. This open porous architecture significantly mitigated mass transfer resistance, propelling the Pb(II) capacity to 486 ± 28.5 mg/g, respectively. The adsorption kinetics were governed by both surface interactions and pore diffusion, underscoring the pivotal role of structural engineering in maximizing adsorption performance.

However, high adsorption capacity does not represent the ultimate goal of material design; sluggish adsorption kinetics often constitute a bottleneck hindering practical implementation. Addressing this limitation, Sun et al. [62] engineered a chitosan/cellulose phosphonate system via phosphorylation and the Mannich reaction. This contains an adsorption mechanism in aqueous solution, carrying targeted pollutants such as Pb(II) and Cu(II), which have been dispersed in bulk water, followed by two consecutive mechanisms: (1) electrostatic attraction and (2) surface complexation, as delineated in (Figure 2b). The synergistic interplay between phosphonate moieties and amino groups proved pivotal in optimizing the adsorption process. This coordination enabled a significant kinetic breakthrough, reducing the equilibrium time to under 10 min while maintaining a robust capacity of 211.42 mg/g. Analogous precision tailoring of functional groups was demonstrated by Xing et al. [63]. By grafting polyethyleneimine (PEI) onto TEMPO-oxidized cellulose to introduce high-density amine active sites, they not only endowed the material with broad-spectrum affinity for Pb(II) (279.32 mg/g) and Cu(II) but also significantly ameliorated both adsorption selectivity and kinetic rates. In addition, the effect of the adsorption capacity of XMene cellulose composite hydrogel is illustrated in (Figure 2c).

Translating bench-scale findings into industrial reality requires overcoming specific technical hurdles. Foremost among these is the need to ensure the adsorbent’s long-term cyclic stability and its efficiency in solid–liquid separation. Addressing the stability issue, Zhang et al. [64] developed an rGO-CMC composite hydrogel that exhibited not only substantial Pb(II) uptake (184.9 mg/g) but also broad-spectrum capabilities toward multiple heavy metals (e.g., Cu(II), Ni(II)). Underpinned by unique ion-exchange and chelation mechanisms coupled with superior mechanical stability, the material retained a removal efficiency exceeding 90% after five cycles of reduced graphene oxide/carboxymethyl cellulose. The rGO-CMC composite was regenerated by immersing Pb(II)-loaded adsorbent in 0.1 M HNO_3_ for 0.5 h, then washing to neutral, highlighting the pivotal role of structural integrity in ensuring long-term reusability and energy storage applications. Furthermore, to mitigate the substantial energy footprint inherent in traditional filtration and centrifugation, researchers have turned to a novel solution: designing materials with intrinsic stimuli-responsiveness. Zhou et al. [65] synthesized magnetic hydrogel beads (m-CS/PVA/CCNFs) via the instantaneous gelation of carboxylated cellulose nanofibers (CCNFs) and amine-functionalized magnetic nanoparticles within a PVA/chitosan matrix. Benefiting from abundant surface carboxyl groups and well-dispersed magnetic nanoparticles, this composite achieved rapid separation induced by an external magnetic field and efficient regeneration (>90% efficiency over four cycles; the reusability of m-CS/PVA and m-CS/PVA/CCNFs hydrogels was tested with 0.01 M HNO_3_), while sustaining a high capacity of 171.0 mg/g (Langmuir model). Taken together, the data suggest a bright future for cellulose-derived hydrogels in the remediation of challenging wastewater streams. This viability is fundamentally underpinned by the material’s ability to combine exceptional structural robustness with unwavering operational consistency.

### 4.2. Adsorption of Cu(II)

Based on the consensus that Cu(II) tends to form stable inner-sphere complexes with oxygen- and nitrogen-containing functional groups, the research paradigm for cellulose-based hydrogels has shifted significantly: from developing single-component materials to multidimensional composite and functionalization designs that maximize the availability of such binding sites. To overcome practical limitations such as limited adsorption capacity, challenging solid–liquid separation, and high costs, current research strategies focus on three primary directions: first, enhancing adsorption kinetics and capacity by constructing interpenetrating or dual-network structures and introducing synergistic effects through nanomaterials [66,67,68]; second, imparting magnetic responsiveness to materials by embedding functional units like magnetic nanoparticles, enabling rapid; and third, low-energy solid–liquid separation, and realizing low-cost, green manufacturing by exploiting agricultural waste valorization pathways [69,70,71].

**Figure 3:**
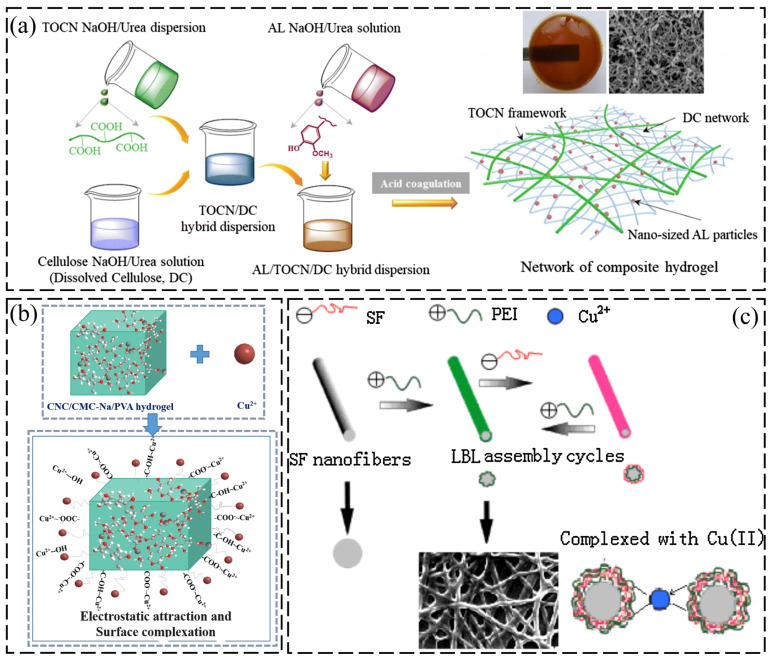
(**a**) Preparation of AL/TOCN/DC composite hydrogel. Reproduced with permission from reference [67]. Copyright 2018, Springer Nature. (**b**) Study on the synergistic mechanism between CNC/CMC-Na/PVA hydrogel and Cu(II). Reproduced with permission from reference [68]. Copyright 2022, Springer Nature. (**c**) Schematic representation of copper ion removal from aqueous solution. Reproduced with permission from reference [71]. Copyright 2015, Elsevier.

Precise regulation of crosslinked networks and the targeted incorporation of functional components constitute the core design principles for achieving an optimized balance between hydrogel swelling characteristics and the number of adsorption sites. Teow et al. [66] systematically investigated the impact of crosslinker dosage, revealing that cellulose hydrogels with a lower crosslinking degree (6 *v*/*v*%) possessed a looser porous architecture. This structure significantly mitigated the internal diffusion resistance for Cu(II), resulting in an adsorption capacity of 28.4 mg/g. To further surpass adsorption limits, the Interpenetrating Polymer Network (IPN) strategy has been employed to engineer sophisticated topological structures. Zhang et al. [67] constructed an IPN composite hydrogel utilizing TEMPO-oxidized cellulose nanofibers (TOCN) and lignin nanoparticles. Taken together, this (Figure 3) illustrates the fabrication and assembly mechanism of a composite hydrogel system incorporating cellulose, metal nanoparticles, and layer-by-layer (LBL) assembly for heavy metal ion adsorption. For instance, (Figure 3a) shows the composite hydrogel synthesis on the left side; TOCN NaOH/urea dispersion undergoes dissolution and is assembled with AL NaOH/Urea solutions to prepare TOCN/DC hybrid dispersion in a beaker, due to the cellulose NaOH/urea solutions (dissolved cellulose, DC). On the right side, a network of composite hydrogel with interpenetrating TOCN and DC networks is embedded with nanoparticles. Through a synergistic interplay, these components explicitly fortified the mechanical framework while simultaneously enriching the matrix with abundant carboxyl and phenolic hydroxyl sites. This dual enhancement was instrumental in attaining a remarkable adsorption capacity of 541 mg/g. Notably, emerging research has begun to explore a closed-loop paradigm of “adsorption-valorization”. Wang et al. [68] developed a CNCs/CMC-Na/PVA system wherein CNCs significantly enhanced electrostatic attraction and surface complexation, elevating the capacity to 108 mg/g. As shown in (Figure 3b), the top left displays CNC/CMC-Na/PVA hydrogel (cyan/turquoise porous structure) with Cu^2+^ ions, while the bottom left side displays the magnified view showing Cu^2+^ ions interacting with the hydrogel network. This illustrates electrostatic attraction and surface complexation between functional groups -COO^−^, -COOH, and Cu^2+^, respectively. More pioneeringly, they proposed a “treat waste with waste, turn waste into wealth” strategy: directly repurposing the Cu(II)-saturated hydrogel as an efficient catalyst for biomass conversion. This approach offers highly inspiring insights for the high-value utilization of heavy metal pollutants.

In practical engineering scenarios, the efficiency of material recovery is often hindered by difficult separation processes. To overcome this bottleneck, the integration of magnetic susceptibility has become a premier design strategy, facilitating rapid and effective solid–liquid separation. Guided by green chemistry principles, Li et al. [69] developed a superparamagnetic composite hydrogel by incorporating Fe_3_O_4_ nanoparticles into cellulose nanofibers (CNF) derived from soybean residue. Their study confirmed that the dimensional effects of CNF structurally reinforced the material’s swelling and mechanical properties. The perennial difficulty of harvesting adsorbents from heterogeneous aqueous systems is effectively surmounted by this approach. By reacting to an external magnetic field, the material achieves complete separation in mere seconds, streamlining the recovery process significantly. Notably, Li’s work also foreshadows a pivotal trend: the valorization of agricultural waste. To control material production costs, research has primarily focused on utilizing renewable biomass resources such as bagasse and wheat straw as raw materials. Addressing the time-consuming drawbacks of traditional hydrothermal methods, Baiya et al. [70] employed a microwave-assisted approach to rapidly prepare CMC/PVA composite gels from bagasse. Although its adsorption capacity for target pollutants is relatively limited (2.3 mg/g), this microwave-synthesis pathway demonstrates significant advantages in energy efficiency and productivity. Its rapid reaction kinetics and low energy consumption provide crucial process feasibility validation for developing biomass-based, low-cost adsorbent materials. In contrast, strategies such as graft copolymerization and semi-interpenetrating polymer networks (semi-IPN) are more effective in fully exploiting the adsorption potential of biomass. Zhou et al. [71] applied this strategy to modify cellulose-based composite hydrogels for the removal of heavy metals. By incorporating polyethylenimine (PEI) macromolecules and layer-by-layer assembly of SF/PEI nanofibers for enhanced Cu^2+^ complexation and removal efficiency, as mentioned in (Figure 3c), they not only proliferated binding sites (e.g., -NH_2_ and oxygen-containing groups) but also catapulted the Cu(II) adsorption capacity to 59.7 mg/g.

In the context of emergency water treatment, materials must simultaneously demonstrate rapid uptake rates and structural integrity. To satisfy these rigorous demands, research has increasingly turned toward strategies utilizing carrier reinforcement. Niu et al. [72] developed a chitosan/polyethylene terephthalate (PET) composite adsorbent (CCP) utilizing this approach. Through a dip-coating technique, the study achieved the robust immobilization of the biopolymer onto the synthetic fiber surface. Experiments demonstrated that CCP achieves adsorption equilibrium within 60 min and exhibits simultaneous removal capabilities for heavy metal ions, including Pb(II), Zn(II), and Cu(II), with adsorption capacities ranging from 18 to 32 mg/g. Benefiting from its low-cost preparation process and macroscopic morphology suitable for open water bodies, CCP also shows promising application potential as an emergency adsorption barrier in complex water environment remediation. Regarding cellulose cryogels, their 3D interconnected macroporous, sponge-like architecture makes them highly effective for emergency heavy metal removal because contaminated water can rapidly flow through their large pores. For large-scale separation, they offer a unique advantage: easy regeneration achieved by simply squeezing the elastic cryogel, thereby avoiding energy-intensive centrifugation. A representative example is presented by Zhang et al. [73], who masterfully bridged the microscale and macroscale by utilizing micro-fibrous cellulose (MFC) as an enhancer. They addressed the problem that vinyl imidazole (VIM) monomers cannot form stable cryogels alone by proposing a poly(VIM)/MFC cryogel matrix. The resulting material possesses interconnected pores with an average size of (12 ± 10 μm), demonstrating a wide pore size distribution. Notably, after 200 underwater compression cycles, the energy loss coefficient remained below 23%, demonstrating extraordinary shape recovery and fatigue resistance. Separately, for chemical regeneration, the Cu(II) saturated cryogel was desorbed using 1 M HCl and then regenerated by washing with deionized water. Batch experiments showed maximum adsorption capacities of 87.4, 53.9, 48.6, 44.3, and 23.8 mg/g for Cu(II), Pb(II), Zn(II), Cd(II), and Ni(II), respectively. In addition, such data fit well with the pseudo-second-order kinetic model and Langmuir isotherm model, delineating monolayer chemisorption. Furthermore, formatting bulk cellulose gels into micro/nanoscale particulate structures demonstrates another highly effective strategy. Their miniaturized dimensions essentially eliminate intraparticle diffusion resistance, exposing more accessible binding sites and thereby providing extremely fast adsorption kinetics compared to traditional bulk hydrogels [74].

### 4.3. Adsorption of Cd(II)

The “soft acid” nature and relatively large ionic radius of Cd(II) impose stringent demands on the pore architecture and coordination potential of adsorbents. This creates an intrinsic thermodynamic mismatch with the “hard base” oxygen-containing groups inherent to the cellulose backbone, which preferentially bind to hard acid cations. The inherent porous network of cellulose provides excellent mass transfer pathways, facilitating efficient pollutant diffusion. However, in complex aquatic environments where multiple ions coexist, the native surface functional groups of cellulose are susceptible to interference from competitive ions, leading to reduced selective adsorption capacity. Consequently, the targeted introduction of multiphase composite components and specific recognition groups guided by the Hard–Soft Acid–Base (HSAB) theory has emerged as an effective strategy for enhancing material selectivity and binding affinity [75,76,77]. Currently, research in this field has shifted its focus from solely pursuing high adsorption capacity toward a multifunctional integrated design paradigm. This approach aims to simultaneously overcome key engineering bottlenecks in material applications—such as selective recognition, interference resistance to complex matrices, and recyclability—through multidimensional structural regulation [78,79,80].

To overcome the inherent limitations of single-component hydrogels in adsorption performance, developing multiphase composite structures has become the core approach for enhancing materials’ broad-spectrum removal capabilities and selective recognition performance toward multiple pollutants. In the realm of organic–inorganic hybrids, Wong et al. [75] embraced the “waste-control-by-waste” paradigm by incorporating nano-hydroxyapatite derived from clam shells into a cellulosic matrix. This composite demonstrated robust anti-interference resilience during the remediation of compositionally complex palm oil mill effluent (POME), achieving the simultaneous sequestration of five coexisting ions (Cu, Pb, Fe, Cd, and Zn). Conversely, within all-organic polysaccharide frameworks, Hameed et al. [76] utilized the crosslinking of carboxymethyl cellulose with potato starch. By leveraging the synergistic interplay between natural polysaccharide chains, they established a preferential adsorption hierarchy of Cd(II) > Pb(II) > and Fe(II), providing a thermodynamic rationale for the separation of specific heavy metals. Moving towards molecular precision, Kundu et al. [77] introduced hydrophobic cavity-bearing β-cyclodextrin to construct a host-guest recognition system. Although the adsorption capacity of this material for the target pollutant (26.42 mg/g) is moderate, its significance lies in successfully demonstrating the feasibility of achieving highly selective adsorption through a mechanism based on inclusion complex formation.

In this field of research, breakthrough progress relies not only on the precise control of adsorption properties at the molecular level, but also on the controlled construction of macroscopic material structures and the development of environmentally friendly preparation processes. These physical and environmental considerations ultimately dictate whether a high-performance material can be successfully deployed in the field. To facilitate operational feasibility and efficient recovery, research attention has increasingly pivoted towards hydrogel films and sustainable solvent systems. In terms of morphological configuration, Ayouch et al. [78] engineered highly transparent CMC-HEC hydrogel films crosslinked by citric acid. This architecture facilitates/enables facile solid–liquid separation, coupled with a superior Cd(II) adsorption capacity of 126.58 mg/g. Focusing on solvent innovations, Brião et al. [79] achieved a breakthrough by utilizing a novel solvent system (CUEN) to fabricate sisal fiber hydrogels. Through this approach, the material is engineered to possess an extraordinary swelling ratio exceeding 5000%, without compromising its ability to trap heavy metals via functional group complexation. This strategy effectively reconciles massive volume expansion with precise chemical capture. The maximum adsorption capacity for Cd(II) is 0.41 mmol/g.

The practical significance of adsorption technologies is predicated on their engineering scalability and their integration into resource valorization frameworks. Transitioning from static batch experiments to continuous flow dynamics, Heidarzadeh-Samani et al. [80] advanced laboratory findings to a pilot-scale dimension by constructing a fixed-bed column (FBC) system based on CNFs/St-g-PAA. Under optimized hydraulic parameters (pH 5, 10 mg/L inflow, 5 mL/min flow rate), the system achieved a removal efficiency of 82.5%. The adsorption process, governed by ionic interactions with carboxylate moieties, conformed to the Langmuir isotherm (40.65 mg/g), while the Thomas model accurately predicted the dynamic breakthrough curves. Notably, the superior regeneration efficiency (>90%) maintained over repeated three cycles attests to the system’s robustness for practical industrial implementation of (Cd (II) removal efficiency of the hydrogel over adsorption–desorption cycles in batch and continuous processes, 0.1 M HCl). Exemplifying the vanguard of “adsorption-coupled-catalysis”, Wang et al. [81] achieved a high capacity of 95.62 mg/g at 313.5 K. Uniquely, the straw cellulose microspheres, upon Cd(II) enrichment, were in situ transformed into CdS nanophotocatalysts. This research marks a paradigm shift from traditional passive pollutant sequestration to active catalytic upgrading. Its core lies in transforming toxic waste into high-value materials with visible-light activity, signifying a crucial step forward in resource recycling and regeneration technology.

### 4.4. Adsorption of As(III)

In most natural aquatic systems (pH < 9.2), arsenic predominantly exists as As(III) in the electrically neutral form H_3_AsO_3_—a speciation that creates remediation challenges far more complex than those posed by As(V). The absence of net charge fundamentally undermines conventional adsorption strategies that rely on electrostatic interactions. Addressing this limitation demands a departure from traditional physisorption approaches toward engineered, multifunctional adsorbent architectures. This strategy entails the following: (1) tailoring active sites for the specific recognition of neutral species (e.g., via coordination complexation) [82,83], and (2) simultaneously optimizing mass transfer kinetics while bolstering structural robustness to endure complex aqueous matrices [84,85].

**Figure 4:**
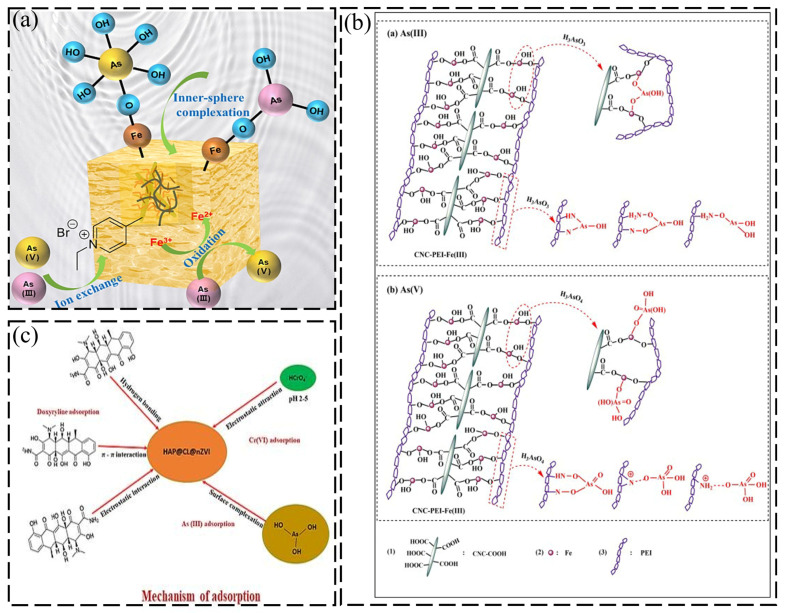
(**a**) Schematic illustration of the arsenic removal mechanism by FN@CPqP hydrogel. Reproduced with permission from reference [82]. Copyright 2024, Elsevier. (**b**) Schematic illustration of the adsorption mechanism of As(III) by CNC-PEI-Fe(III). Reproduced with permission from reference [83]. Copyright 2020, Elsevier. (**c**) Schematic illustration of the possible adsorption mechanisms for doxycycline, Cr(VI), and As(III) removal by HAP@CL@nZVI. Reproduced with permission from reference [87]. Copyright 2024, Elsevier.

The integration of magnetic nanomaterials with cellulose hydrogels enables the construction of a dual-functional material platform that combines highly efficient adsorption with convenient magnetic recovery capabilities. Su et al. [82] engineered a tailored FN@CPqP hydrogel by integrating quaternary ammonium moieties and compositing CNC with magnetic Fe_3_O_4_ nanorods (as depicted in Figure 4a). The homogeneous distribution of nanorods throughout the gel matrix prevented particle agglomeration while promoting the formation of a well-defined three-dimensional porous network. This structural advantage translated directly into performance gains: the composite’s adsorption capacity for As(III) surged from 241.3 mg/g to 263.0 mg/g compared to the pristine hydrogel. Notably, it achieved nearly 95% pollutant sequestration at neutral pH with a minimal dosage (0.5 g/L). Kinetic analysis confirmed the process as typical monolayer chemisorption, with the material exhibiting robust stability over five regeneration cycles (the hydrogels were regenerated with 0.1 M NaCl-NaOH, rinsed to pH 7). Diverging from the reliance on physical magnetism, Xi et al. [83] exploited the chemical “bridging” potential of iron ions. Fe^3+^ was utilized as a crosslinker to bridge CNC and PEI, resulting in the formation of a dense network rich in O-Fe-O linkages (as depicted in Figure 4b). These in situ formed chemical bonds demonstrated exceptional affinity for arsenic species, yielding capacities of 142.42 mg/g for As(III) and 78.71 mg/g for As(V). Crucially, the valence state proved pivotal—experiments confirmed that only Fe(III) possesses this specific bridging capability.

To simultaneously address the challenges of continuous processing and material recovery in industrial settings, research focus is shifting toward developing structured materials such as hydrogel membranes with a high specific surface area. Karbassiyazdi et al. [84] engineered a composite hydrogel membrane based on cellulose acetate (CA) and carbon-based aluminum hydroxide. This material demonstrated impressive broad-spectrum purification capabilities, achieving removal efficiencies of 99.9% for As(III) and over 96% for heavy metals such as Cu(II) and Pb(II). This membrane structure demonstrates excellent practical potential through its efficient solid–liquid separation design, achieving up to four regeneration cycles while maintaining a retention rate exceeding 98% (in 0.1 M Al_2_(SO_4_)_3_ for 30 min, followed by rinsing with 0.1 M NaHCO_3_ until the pH returned to neutral). Building upon this foundation, integrating metal-organic frameworks (MOFs) to construct composite structures has emerged as a key strategy for enhancing the material’s overall performance in pursuit of higher pollutant loading capacity. Al-Hazmi et al. [85] employed epichlorohydrin crosslinking to successfully embed a tryptophan-modified Yttrium-based MOF (NH_2_-Y-MOF) within a chitosan/carboxymethyl cellulose network. The MOF’s unique topology enabled the composite to achieve a substantial specific surface area of 864 m^2^/g and a well-defined pore size of 1.38 nm. This microstructural metamorphosis propelled the saturation adsorption capacity for As(III) to a remarkable 326.3 mg/g. Thermodynamic and kinetic analyses characterized the adsorption as a spontaneous, endothermic process driven by the synergy of surface complexation, electrostatic interactions, and hydrogen bonding. The high stability, demonstrated over eight cycles, establishes the composite as a competitive candidate for next-generation high-efficiency adsorbents.

Beyond chemical moiety modification, the “nano-confinement effect,” grounded in physical spatial modulation, opens a novel dimension in adsorbent design. Li et al. [86] proposed an ingenious strategy that employs a simple “dehydration-rehydration” cycle to induce irreversible structural shrinkage in iron hydrate-loaded cellulose beads (FCB). This process precipitated pore reorganization, successfully constructing a cellulose-confined iron hydrate (CCF) system. This microstructural remodeling significantly elevated the As(III) adsorption capacity from 117.26 mg/g to 169.25 mg/g. Impressively, the material exhibited an ultra-long service life (>4000 bed volumes) in dynamic column assays. Furthermore, the spent adsorbent complied with TCLP toxicity leaching standards, striking an optimal balance between high efficiency and environmental safety. Addressing the challenge of co-existing pollutants in complex environmental matrices, waste-treating-waste and multi-functional synergy design strategies have gained increasing prominence. Kotnala et al. [87] valorized eggshell waste into hydroxyapatite (HAP) and integrated it with cellulose and nano zero-valent iron (nZVI) to fabricate a ternary composite, HAP@CL@nZVI. This green bionanocomposite transcended the limitations of single-pollutant remediation. Governed by pH-dependent kinetics, it achieved the simultaneous, high-efficiency removal of As(III) (79.37 mg/g), antibiotics (Doxycycline), and heavy metals (Cr(VI)) through a flexible interplay of electrostatic attraction, π-π stacking, and surface complexation.

The mechanism of As(III) removal by Fe_3_O_4_NRs@CNC-g-PAA/qP4VP hydrogel can be mentioned in (Figure 4a). The pH experimental results show that the interactions between Fe_3_O_4_NRs@CNC-g PAA/qP4VP hydrogel and arsenic were mainly the formation of electrostatic interactions between the positive quaternary ammonium salts of qP4VP and negative As(III) ions (i.e., H_2_AsO_3_) in water. Also, the presence of a large number of O–Fe–O bonds in Fe_3_O_4_NRs in the hydrogel forms incredibly stable Fe–O–As bonds with arsenic ions. We used the “bridge joint” effect of iron ions; cellulose nanocrystal-containing high-performance adsorbents were synthesized via a coprecipitation scheme, which enriched the crosslinking action of cellulose nanocrystal and polyethyleneimine. Based on the evidence, the iron ions effectively attach the two dispersed polymers, inducing a great number of O-Fe-O bonds and preparing more adsorption active sites for the elimination of extremely contaminated and highly toxic As(III)/As(V), as delineated in (Figure 4b), respectively. On the other hand, (Figure 4c) shows a biogenic nanocomposite (HAP@CL@nZVI) had been positively established using hydroxyapatite derived from cellulose, and green-synthesized nano zero-valent iron for the elimination of Cr(VI), and As(III) from aqueous solutions through the action mechanism of adsorption.

### 4.5. Adsorption of Cr(VI)

Unlike As(III), which remains electroneutral in acidic media, Cr(VI) is present predominantly in anionic forms (specifically HCrO_4−_ or CrO_4_^2−^) within the pH range of 2–6. This electronegative characteristic induces a specific electrostatic repulsion (the Donnan exclusion effect) against the negatively charged surface of pristine cellulose, establishing a formidable thermodynamic barrier to the adsorption process. To surmount this bottleneck, early strategies primarily relied on monomer grafting to introduce reactive functional sites. While Truong et al. [88] demonstrated the feasibility of Cr(VI) removal via carboxyl sites through the copolymerization of acrylic acid and cellulose, the modest adsorption capacity of 4.14 mg/g exposed the inherent limitations of mono-functionalization strategies in overcoming strong electrostatic energy barriers. Consequently, contemporary research has pivoted from simple surface modification towards constructing holistic modification systems. These advanced frameworks integrate interfacial charge modulation, microstructural remodeling, and multifunctional integration to achieve superior performance.

**Figure 5:**
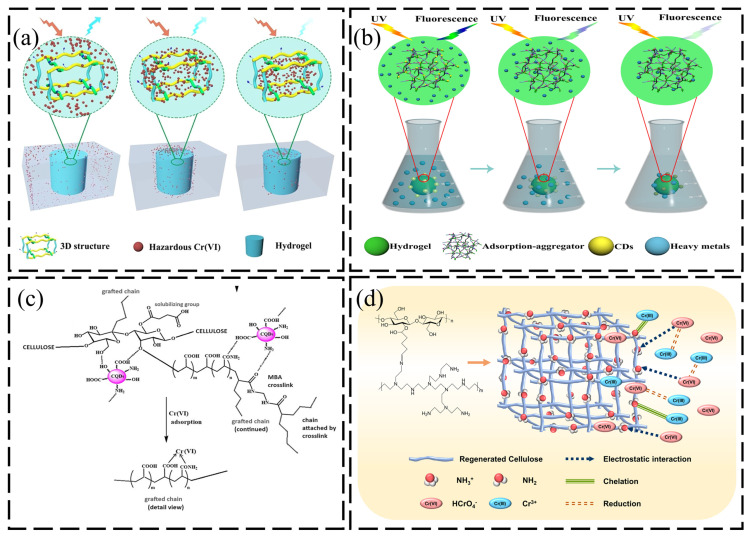
(**a**) Mechanism of Cr(VI) adsorption and detection by hydrogels. Reproduced with permission from reference [89]. Copyright 2021, MDPI. (**b**) Mechanism of FLH-6 for efficient adsorption and detection of Cr(VI). Reproduced with permission from reference [90]. Copyright 2021, Elsevier. (**c**) Ion exchange mechanism between CS-g-poly(AM-co-AA) and Cr(VI). Reproduced with permission from reference [91]. Copyright 2024, Springer Nature. (**d**) Mechanism of Cr(VI) adsorption and reduction by P/RC. Reproduced with permission from reference [93]. Copyright 2022, Elsevier.

Smart nanocomposites that integrate in situ detection with high-efficiency sequestration represent the vanguard of adsorbent engineering. Zeng et al. [89] ingeniously leveraged carbon dots (CDs) into the CS/CNCD composite as dual-functional components, serving simultaneously as fluorescent probes and auxiliary adsorption sites. Based on the review literature and data analysis, the CS/CNCD composite hydrogel’s Cr(VI) sorption and detection mechanism can be postulated in (Figure 5a). The Cr(VI) was first augmented into the interface via electric attraction, respectively. After that, Cr(VI) was slowly diffused into the inside along 3D porous structures. In this diffusion process, a substantial amount of Cr(VI) was decreased to Cr(III), and then some of the Cr(III) ions intermingled with the hydroxyl and carboxyl groups. Due to these limited structures, the CS/CNCD composite hydrogel carried a high adsorption capacity. Concurrently, Cr(VI) is also supplemented into the CD via electric attraction, resulting in fluorescence quenching. As supportive evidence, the CS/CNCD composite hydrogel possessed a susceptible detection experience. Driven by the inner filter effect (IFE) and static quenching mechanisms, this system achieved ultrasensitive detection of Cr(VI) (LOD: 0.04 μg/L). Simultaneously, the robust 3D network underpinned by cellulose nanocrystals significantly amplified heavy metal capture (217.8 mg/g). The unit removal cost was approximately 60-fold lower than commercial activated carbon, demonstrating excellent economic viability. To further exploit the ultimate adsorption limits of biomass, Yuan et al. [90] incorporated lignin into the CD/cellulose matrix. The synergy between lignin’s abundant aromatic moieties and modified CDs constructed efficient ion transport channels. The fluorescent lignin-based hydrogel with carbon dots (CDs) was synthesized for the high-level utilization of lignin and control of hexavalent chromium (Cr(VI)). The CDs fixed to the hydrogel offered a quick reaction to Cr(VI) as mentioned in (Figure 5b). This microstructural optimization precipitated a quantum leap in performance, yielding a staggering maximum capacity of 599.9 mg/g. This work not only successfully achieved visual monitoring of the adsorption process, but more importantly, it revealed a key mechanism: in multilayer adsorption systems following the Freundlich model, precise control over the material’s porous structure is the decisive factor in exceeding its theoretical adsorption capacity.

Heteroatom doping has proven effective for enhancing surface active sites and optimizing pore structure in carbon nanomaterials. Employing a microwave-assisted pyrolysis strategy, Tohamy et al. [91] upcycled agricultural waste (bagasse) into highly active nitrogen-doped carbon quantum dots (N-CQDs) and in situ embedded them within an acrylic acid/acrylamide grafted cellulose network. Amino-modified cellulose illustrated the Cr adsorption performance of the exchange mechanism through grafting, achieving a Cr(IV) adsorption capacity higher than 125 mg/g, delineating its strong potential as an adsorbent for water treatment, as shown in (Figure 5c). N-CQDs provided dual functionality: fluorescence sensing for detection and pore structure modification for enhanced adsorption. At 10% loading, induced mesoporous channels facilitated mass transfer, achieving 85.8% Cr(VI) removal efficiency. Kinetic analysis revealed combined physisorption–chemisorption mechanisms, significantly enhancing heavy metal capture. Parallel to functionalized nanomaterials, using pristine biomass as a reinforcing filler represents a cost-effective alternative. To curtail costs and achieve the valorization of biomass resources, Marciano et al. [92] incorporated eucalyptus residues (PER) and their treated derivatives (TER) into a gelatin hydrogel matrix. Through the restructuring of the hydrogen-bonding network, the cellulosic fillers induced significant changes in matrix crystallinity, thereby elevating thermal stability by ~10 °C. While this rigid network constrained swelling—limiting adsorption capacities to the range of 12–13 mg/g—the study demonstrates the viability of hydroxyl-rich biomass residues (specifically TER 5%) in reconciling structural integrity with cost-effective remediation.

Solution regeneration and chemical modification are two key synergistic strategies for regulating the adsorption properties of cellulose materials: the former reshapes their porous structure to optimize mass transfer, while the latter enhances electrostatic attraction to target substances by reversing surface charges. Kim et al. [93] engineered a regenerated porous nanofiber hydrogel (P/RC), distinguished by the cationic grafting of polyethyleneimine (PEI). This modification imparted a strong positive charge affinity to the otherwise inert cellulose backbone and positively elevated the number of active adsorption regions. Taken together, the most common adsorption mechanism for the removal of pollutants and heavy metals is electrostatic interactions; however, combinations of other interactions along with electrostatic interaction can also be reported in chelation, reduction, and adsorption processes (Figure 5d). Experimental data indicated that this interface engineering strategy not only surmounted the mechanical limitations of natural cellulose but also achieved an ultra-high Cr(VI) adsorption capacity (578 mg/g) through the synergistic interplay of electrostatic attraction and chelation. Crucially, the modified regenerated cellulose addressed the issue of structural collapse during long-term operation while retaining high permeability. Furthermore, the ultimate objective of remediation technology is undergoing a paradigm shift from “adsorptive sequestration” to “reductive detoxification.” Pradyasti et al. [94] leveraged polyelectrolyte complexation (PEC) principles to design a ternary biopolymer hydrogel loaded with noble metal nanowires (Pd/Au/Ag/Pt). By exploiting charge matching between polymer chains and a multivariate crosslinking strategy (glutaraldehyde/citric acid/Ca (II)), they constructed a catalytic support with superior mass transfer kinetics. Irregularly shaped nanowires maintained high electrochemically active surface area (42.3 ± 3.1 cm^2^) within the PEC matrix, enabling 95.2 ± 1.8% Cr(VI) reduction to Cr(III) in continuous flow (residence time = 15 min, flow rate = 30 mL/min). This work not only substantiated the viability of natural polymers as stabilizers for metallic nanomaterials but also provided a blueprint for next-generation environmental materials integrating “adsorption-reduction-detoxification.”

### 4.6. Adsorption of Rare Metal Ions

Driven by the rapid expansion of nuclear energy and high-tech industries, the selective recovery of rare and strategic metal ions from complex aqueous systems has become a critical global challenge. Particularly for rare radioactive metals like uranium, traditional cellulose hydrogels often exhibit subpar mechanical stability. To overcome this limitation, Zhang et al. [95] employed radiation-induced graft polymerization to construct a robust dual-network hydrogel. After modification with phytic acid, the hydrogel achieved a U(VI) adsorption capacity of 449.5 mg/g, attributed to the synergistic complexation of phosphate and carboxyl groups. To push the boundaries further for sustainable uranium recovery, Xu et al. [96] engineered an amidoxime-functionalized carboxymethyl cellulose (CMC)/MXene composite hydrogel. In addition to achieving an exceptional U(VI) capture capacity of 549.4 mg/g, the composite also exhibited rapid desorption (completed in 3 h using 0.03 M HCl) and outstanding reusability, maintaining ≥93% adsorption and ≥89% desorption after five cycles. Intriguingly, this design introduced an enzyme-triggered degradation pathway, which enables benign depolymerization of the matrix, offering an eco-friendly alternative to incineration. Similarly, to target radioactive metals, Sherbiny et al. [97] incorporated sugarcane bagasse-derived cellulose nanocrystals into an irradiated Acacia gum/acrylamide matrix, which significantly improved structural integrity and achieved an Sr(II) capacity of 370 mg/g.

In addition to radionuclides, functionalized cellulose gels also exhibit tremendous potential for the selective recovery of indispensable energy metals. For instance, Wang et al. [98] utilized 3D direct-ink-writing technology to fabricate a shape-programmable, macroporous hydrogel integrating CMC, sodium alginate, and exfoliated montmorillonite nanosheets. Driven by interfacial complexation and cation exchange, this chemically robust matrix demonstrated excellent selectivity and scalability in continuous fixed-bed operations, achieving a Ga(III) capture capacity of 74.42 mg/g. Meanwhile, extracting Li(I) from salt lake brines is notoriously challenging because the strongly alkaline environments cause severe equipment corrosion. To address this, Liu et al. [99] devised an ingenious quaternary ammonium (QA)-functionalized CMC hydrogel loaded with H_2_TiO_3_ (HTO) lithium-ion sieves, designed to withstand strongly alkaline conditions. The grafted QA groups acted as an in situ pH buffer to neutralize released protons, thereby enabling efficient Li(I) extraction (18.17 mg/g—a 142% enhancement over pure HTO powder) under mild, weakly alkaline conditions (pH = 8). Simultaneously, the hydrogel format perfectly resolves the solid–liquid separation challenges associated with loose powder adsorbents.

## 5. Conclusions and Perspectives

In summary, cellulose-based composite hydrogel has become extensively researched owing to its excellent adsorption capacity, non-toxic and environmentally friendly qualities, biodegradability, and renewability, offering a promising foundation for industrial wastewater treatment. Recent advancements have revealed that cellulose-based composite hydrogels possess excellent capabilities for heavy metal remediation, environmental purification, and adsorption mechanisms. Through targeted alteration, cellulose-based composite hydrogels have alleviated the remediation of pollutants from wastewater, sustainable applications, and significant potential in controlling heavy metal contamination.

Instead of prominent progress, significant tasks remain before these materials may realize broader-scale, easy to handle, and practical implications with suitable authenticity. We noticed that most current findings were limited to idealized lab-based circumstances that could not reflect the actual-world biosphere containing water contamination, heavy metal pollutants, textile industry waste, mining drainage, agricultural runoff, water chemistry, ion compartmentalization, and competing environmental effects. To our knowledge, such a discrepancy generates a potential gap between field conditions and lab-based consequences, underscoring the urgent necessity for global evolution under eco-friendly circumstances. Altogether, studies have generally concentrated on physical crosslinking, chemical grafting, supramolecular self-assembly, control of microscopic pore structures, macroscopic mechanical strength, and diverse synthesis techniques, leaving many abundant substitutions unexplored and limiting the adsorption of multifunctional applications of composite hydrogels. The research focused on heavy metal remediation efficiency has often been overshadowed by emergent attentions, for instance, large-scale adoption in real water bodies, natural variability, sequestering cationic heavy metals, removal of pollutants from water bodies, and sustainable post- as well as pre-treatment roadmaps.

Other methods use physical, chemical, and grafting techniques; interactions, self-assembly, microscopic pore structures, and macroscopic mechanical strength; as well as inorganic synergistic nanomaterials, Hard–Soft Acid–Base (HSAB) theory, adsorption–reduction–detoxification, and in situ spectroscopic methods. These can all affect metal ion adsorption due to their abundant modifications. However, it is still rare to find comprehensive studies on the examination of these composite hydrogels for the removal of heavy metals under critical conditions. Based on the literature review, future research directions could adopt an overall approach to address such critical acceptance.

## Data Availability

The original contributions presented in this study are included in the article. Further inquiries can be directed to the corresponding author(s).
